# Chew on This: Persistent Organic Pollutants May Promote Insulin Resistance Syndrome

**DOI:** 10.1289/ehp.118-a173b

**Published:** 2010-04

**Authors:** Tanya Tillett

**Affiliations:** **Tanya Tillett**, MA, of Durham, NC, is a staff writer/editor for *EHP*. She has been on the *EHP* staff since 2000 and has represented the journal at national and international conferences

Animal studies indicate some persistent organic pollutants (POPs) may be endocrine disruptors and suggest similar health risks for humans. Recent studies have further suggested an association between exposure to POPs and the prevalence of type 2 diabetes. Now an experimental animal study reports evidence of a causal link between POP exposure and insulin resistance syndrome, a cluster of metabolic disorders—including type 2 diabetes—that are marked by sustained high blood sugar **[*EHP*** **118:465–471; Ruzzin et al.]**.

POPs accumulate in fatty tissue, where they can remain for years because they are not easily broken down. Fatty fish are a potential source of POP exposure in many human populations. However, *n*-3 polyunsaturated fatty acids in fish oil may have beneficial health effects, possibly including protective effects on insulin resistance, that could counterbalance any adverse effects of POPs in fatty fish.

In the current study, rats were exposed for 28 days to high-fat diets that contained either crude fish oil (from farmed Atlantic salmon) or fish oil that was refined to remove POPs. As expected, the crude fish oil contained much higher levels of POPs than the refined oil. Gene expression profile comparisons of the livers of the 2 treatment groups showed that POP exposure disrupted lipid homeostasis.

Rats on the unrefined fish oil diet gained more weight overall and had increased triacylglycerol, diacylglycerol, and total cholesterol levels compared with rats fed refined fish oil. They also showed impaired insulin action in response to the high-fat diet, whereas the high-fat diet did not seem to cause insulin resistance in the rats fed refined fish oil. Further analysis revealed a reduction in the ability of insulin to stimulate glucose uptake in adipocytes treated with a mixture of POPs that was comparable to the mixture of chemicals in crude fish oil. Adipocyte responses to insulin varied with exposures to different mixtures of individual POPs.

The authors conclude that dietary exposure to POPs may be a risk factor for insulin resistance and associated metabolic disorders. Furthermore, the metabolic effects of POP exposures exacerbated deleterious effects of a high-fat diet on rats and appeared to negate protective effects of *n*-3 polyunsaturated fatty acids on insulin resistance.

